# Notes and News

**Published:** 1899-12-09

**Authors:** 


### Notes and News.

(Collected and Communicated)

The Earl of Leicester has made a further donation of £10,000 to the Norfolk and Norwich Hospital.

The small-pox epidemic at Hull is still very serious. Vigorous measures are now being taken to arrest the progress of the disease. The Evan Fraser Hospital, situated without the city boundary, is being utilised for the treatment of cases.

At the annual students' dinner of the National Dental Hospital it was remarked that the general condition of teeth was worse than formerly. Defective teeth render a large number of recruits ineligible for service in the army.

A corrugated iron hospital is to be erected near Halifax for the reception of small-pox cases. The small hospital now in use will shortly be unavailable, and the iron building is to serve as a temporary measure, whilst the Town Council of Halifax are considering the expediency of erecting a permanent institution.

A report comes from Philadelphia of the death of a patient in one of the city hospitals from burns received while endeavouring to free his limbs from the intense heat of a hot-air apparatus, with which he was being treated. It is said that the patient, a sufferer from rheumatism, becoming alarmed at the heat, kicked the apparatus from the hands of the attendant and upset it upon himself, with the result that his clothes caught fire and he was so burned that he died some days afterwards.

It may surprise many people to learn that Japan possesses specially-equipped hospital ships for use in warfare. These have been provided under the supervision of the Japanese Red Cross Society, which joined the Geneva Convention many years ago. The ships were built in England, and in time of peace are used as passenger boats. The "Kosai-Maru," equipped only last year, has a fine saloon, easily convertible into a ward. Arrangements have been made for infectious cases, and for a distinct sanitary service. Ventilation has been carefully attended to.

The Middlesex Hospital has received £1,000 from Mr. Arthur W. Davis, with authority to the governors to deal with the amount as they deem wisest in assisting the study of cancer. The hospital has for a long time devoted a large amount of accommodation to the treatment of cancer patients.

A new operation theatre has been added to the Evesham Cottage Hospital as a Jubilee memorial. The cost has been £274. The fittings have been paid for out of a fund raised by the matron. The tables and shelves are formed of plate glass, and throughout the theatre care has been taken to secure the latest improvements.

Birmingham is feeling the want of municipal hospital accommodation for typhoid fever and diphtheria. At present the only accommodation available for patients suffering from these diseases seems to be at the two large general hospitals and at the Children's Hospital, and during the recent prevalence of typhoid the numbers received at these institutions have caused considerable inconvenience.

The New York *Medical Record* says that the trustees of the American Medical Association have adopted an official button with which the members are requested to label themselves. It is of gold, with red, white, and blue enamel. In the centre is a lance-pointed cross, the ends of which point to the word MAMA, which is probably not a term of endearment, but indicative of the fact that the wearer is a Member of the American Medical Association.

The League of Mercy, which forms part of the scheme of the Prince of Wales's Hospital Fund for London, has been doing a good work for some time past. A large number of presidents and lady presidents have their organisations complete in the metropolis and home counties. The Prince and Princess of Wales will receive the presidents and lady presidents at Marlborough House on the 18th inst., and will then learn all that has been done on behalf of the Fund.

According to the report recently issued by Surgeon-General Sternberg, of the United States Army, in regard to the health of the American troops, the number of deaths, including regulars and volunteers, from May 1st, 1898, to June 30th, 1899, was 6,619. Of these 496 men were killed in action and 216 by accident, 202 died from gunshot wounds, 2,774 from typhoid fever, 476 from malarial fever, 359 from pneumonia, 342 from diarrhœa and dysentery, and 185 from yellow fever.

The outlook in regard to plague in America is distinctly disquieting. A vessel recently arrived at the New York quarantine station from Santos, in Brazil, with cases of plague on board. Another vessel sailing from Santos, on touching at the Cape de Verde Islands, was found to be infected, three cases having died from plague. At Santos the disease continues to spread, and, in view of the insanitary condition of the town, there seems but little hope of staying its progress. Plague also exists in Rio, both in the town and the suburbs; and at Asuncion, in Uruguay, the disease is reported to be spreading.

